# A66 MICROSCOPIC COLITIS: AN EPIDEMIOLOGICAL ANALYSIS OF MANITOBA’S POPULATION

**DOI:** 10.1093/jcag/gwae059.066

**Published:** 2025-02-10

**Authors:** P Shah, K Al-Bayati, K Parkinson, G Fischer, H Singh, C N Bernstein, S Shaffer

**Affiliations:** University of Manitoba Max Rady College of Medicine, Winnipeg, MB, Canada; University of Manitoba Max Rady College of Medicine, Winnipeg, MB, Canada; University of Manitoba Max Rady College of Medicine, Winnipeg, MB, Canada; University of Manitoba Max Rady College of Medicine, Winnipeg, MB, Canada; University of Manitoba Max Rady College of Medicine, Winnipeg, MB, Canada; University of Manitoba Max Rady College of Medicine, Winnipeg, MB, Canada; University of Manitoba Max Rady College of Medicine, Winnipeg, MB, Canada

## Abstract

**Background:**

Microscopic colitis (MC) is an inflammatory disease of the colon, causing chronic diarrhea, with 2 histologic sub-types, collagenous colitis and lymphocytic colitis. It tends to affect older individuals, with a female predominance. MC is diagnosed in 10% of patients across all age groups, and in 20% in those over the age of 70 among persons undergoing colonoscopy for chronic watery diarrhea. However, there is a paucity of data in Canadian populations, thus we performed a population-based study in Manitoba to assess the prevalence of MC.

**Aims:**

Determine of the prevalence of MC in Manitoba from 2006 - 2018.

**Methods:**

Pathology reports which mentioned MC (or one of the histologic sub-types) were obtained from the Shared Health Pathology Database, which includes a portion of the pathology reports issued in Manitoba for the years 2006 to 2018. These pathology reports were then examined manually to detect true cases of MC. Patient demographics including age, year of diagnosis, and histologic sub-type (collagenous versus lymphocytic) were obtained. Continuous variables were analyzed using means.

**Results:**

9377 pathology reports were initially identified. Of these, 916 people (9.8%) were identified with MC, with 74 having had more than 1 colonoscopy during this time period. Of the 916 cases of MC, 53.5% (490) had collagenous colitis, 44.8% (410) had lymphocytic colitis, and 1.7% (16) did not have a specified sub-type reported. There was a female predominance: collagenous colitis at 83.9%, lymphocytic colitis at 77.1%, and unspecified subtype at 81.3%. The mean age of diagnosis for collagenous colitis, lymphocytic colitis, and unspecified, was 62.0 years old (SD 13.2), 59.8 years old (SD 14.6) and 60.5 years ols (SD 15.6,), respectively. The age range of diagnoses was 18 to 91.

Of the 70 people who had more than 1 colonoscopy during this time period, 4 people initially had a diagnosis of collagenous colitis which was later switched to lymphocytic colitis, 7 had an initial diagnosis of lymphocytic colitis which was later switched to collagenous colitis, and 1 patient had an initial diagnosis of lymphocytic colitis, then switched to collagenous colitis, and later switched back to lymphocytic colitis.

**Conclusions:**

In Manitoba over a 13 year time-period, 916 (9.8% of those where MC was considered) were diagnosed from colonic biopsy. Collagenous colitis was more common than lymphocytic colitis, with a similar age at the time of colonoscopy, along with both having a female predominance. Further research is needed to understand whether the clinical courses of collagenous and lymphocytic colitis differ, and how each sub-type responds to conventional treatment.

**Funding Agencies:**

**None**

